# Prioritisation of Clinical Research by the Example of Type 2 Diabetes: A Caregiver-Survey on Perceived Relevance and Need for Evidence

**DOI:** 10.1371/journal.pone.0032414

**Published:** 2012-03-20

**Authors:** Stefan Kamprath, Antje Timmer

**Affiliations:** 1 Department of Cardiology, Herz-Zentrum Bodensee, Konstanz, Germany; 2 Department of Clinical Epidemiology, Bremen Institute of Preventive Research and Social Medicine, Bremen, Germany; Universidad Peruana Cayetano Heredia, Peru

## Abstract

**Background:**

The Cochrane Collaboration aims at providing the best available evidence for interventions in health care. We wished to examine to which extent treatments considered relevant by caregivers in type 2 diabetes are covered by Cochrane systematic reviews.

**Methodology/Principal Findings:**

130 different interventions in type 2 diabetes were identified based on a review of clinical practice guidelines and expert opinion (Table S1). 459 members of the German Diabetes Society (diabetologists, general practitioners, diabetic nurses, nutritionists, podologists, others) were surveyed via e-mail-list to rank a) the perceived clinical relevance and b) the perceived need for evidence of interventions, based on an internet survey. In the Cochrane Library, there were, at the time of this evaluation, 56 reviews on interventions in diabetes. Generally, coverage of topics by Cochrane reviews reflected the perceived clinical relevance and perceived need for evidence. As an example, highly ranked treatments such as lifestyle changes or oral antidiabetics were well covered, while low rank treatments such as complementary approaches were not covered. Discrepancies occurred with new treatments such as amylin-analogues (low relevance, high need for evidence, review not yet completed) and interventions with immediate and dramatic effects such as treating hypoglycemia (high relevance, low need for evidence, no review). Also, there was a relative scarcity of reviews concerning specific problems, in particular, treatment of late diabetic complications.

**Conclusions/Significance:**

For most interventions, perceived relevance and perceived need for evidence are reflected by the evidence already available. Prioritizing should aim at improving immediacy and consideration of the treatment of complications.

## Introduction

Clinical research ideally strives to improve the care of patients. The transfer of knowledge from research to care givers and health care policy makers is a necessary prerequisite for this to happen. The mechanisms, quality and determinants of knowledge transfer have, in consequence, attracted quite some research activity [1]–[3]. Various projects and organizations aim to improve dissemination of research results, best known, possibly, the Cochrane Collaboration [4]–[6].

The Cochrane Collaboration was founded in 1993 with the aim to ease access to clinical trial results. Since then, more than half a million citations to clinical trials have been assembled in the Cochrane Database of Clinical Trials, and more than 3000 systematic reviews have been prepared for specific health care interventions in the Cochrane Database of Systematic Reviews. These databases are published in the Cochrane Library which is now considered one of the most important sources of evidence in health care.

The success of the Cochrane Collaboration is based on the enthusiasm of individuals. Reviews are prepared on a voluntary basis by author teams with an interest in a particular research question, supported by topic-specific editorial teams. The Cochrane Collaboration so far had no central board regulating the choice of topics.

Finding ways to prioritize topics for systematic reviews is now among the major concerns of the Cochrane Collaboration, as funds are restricted and the collaboration aims at higher professionalization [7]. It has been estimated that the current content covers about one third of all systematic reviews required [8]. This calculation was based on comparing the average number of clinical trials per review to the overall number of controlled clinical trials identified. There are a few studies looking into ways to prioritize research topics, e.g., from the field of public health [9], emergency medicine and trauma [10], [11], nursing [12], or urinary incontinence [13]–[15], or using a more general approach such as in the Database of Uncertainties of the Effects of Treatments in the UK (http://www.library.nhs.uk/duets/) [16].

We aimed to specifically examine how well therapeutic interventions of important health problems are covered in the Cochrane Library. In particular, we wished to know whether the priorities set by individual contributors reflect the degree of clinical relevance as well as need for evidence perceived by professionals involved in the care of these patients. Type 2 diabetes was chosen as an example, as this is a disease of high global importance. Worldwide, about 347 million people are affected, based on a global adult prevalence of almost 10% in 2008 [17]. The WHO estimates that current numbers may be expected to double until the year 2030. Diabetes-related health care expenses are reported to range from 2.5% to 15% of annual health care budgets and will rise to pose a relevant economic challenge, in particular to middle- and low-income countries [18]. Against the background of limited financial resources an early and effective treatment with a focus on preventable complications remains essential and a constant issue of the quality of health care.

## Materials and Methods

The study was performed as a combination of an internet survey of care givers and literature-based data extraction.

### Item generation, identification of relevant interventions

Interventions were defined as any procedure or process intended to improve or change the health status of an individual with type 2 diabetes. Indications included, for example, glycemic control, prevention of diabetes-specific complications, treatment of complications, improvement of quality of life and management or treatment of common co-morbidities. A list was created based on the clinical situations covered by recommendations in clinical practice guidelines (included as supplemental material). These were identified using the international collection of clinical practice guidelines compiled by the German Physician Center for Quality in Medicine (ÄZQ, Berlin, www.aezq.de). In addition, Medline was searched via PubMed (diabetes (MeSH and text word) in combination with the subset “systematic reviews”, the publication type “clinical practice guideline”, the text word “consensus” and the text word “guideline”), as well as internet sites for guidelines (Guidelines International Network (G-IN), Scottish Intercollegiate Guidelines Network (SIGN) and German Association of the Scientific Medical Societies (AWMF)). The resulting list of 130 different interventions was grouped as follows (Table S1):

Lifestyle modification, interventions aiming at weight reduction

Complementary and alternative medicine interventions (CAM)

Oral antiglycemic medication (classical, new preparations)

Insulin therapy

Treatment of co-morbidity (dyslipidemia, cardiovascular, neurological/psychiatric)

Treatment of late diabetic complications (diabetic foot, peripheral neuropathy, kidney, eye)

Treatment of acute diabetic emergencies

Perioperative management

Complex interventions and specialized care

### Internet survey

An internet questionnaire was developed using the SelectSurvey Software (Version 1.5.4, ClassApps). For each intervention, probands were asked to rate a) the clinical relevance of this intervention and b) the need for external evidence for this intervention, given that common indications for the respective treatment are present. Answers were to be given on a 5-point scale from extremely important to not important at all (or unknown). An additional question probed on general attitudes towards and experience in evidence-based medicine by enquiring on the frequency of use of different resources, such as the Cochrane Library or PubMed.

The questionnaire was piloted among a diverse group of six care givers (one diabetes specialist, one clinical pharmacologist, one diabetic nurse, one endocrinologist, two general practitioners). No interventions were reported to be missing or redundant. As the overall workload for the respondents was felt to be too high, the questionnaire was divided into two parts: a) clinical relevance, b) perceived need for evidence. Both parts of the questionnaire covered the full set of interventions in the same order and grouping.

The link to the survey was mailed to all members of the German Diabetes Society (Deutsche Diabetes Gesellschaft, DDG). This society is the major association of care givers in diabetes in Germany and includes health care professionals of any discipline, such as physicians, both specialized and general, researchers, psychologists, nurses, physiotherapists, podologists, and nutritionists. The exact number of persons on the e-mail list was not available, but is estimated to be around 2000, including an unknown proportion of invalid addresses. There was also no information on the demographic characteristics and specializations of the list participants. Therefore, differences in the response status of the list participants could not be ascertained.

Probands were asked to throw a coin and then open either part 1 or 2 of the questionnaire, depending on the result of the thrust. The titles of the parts of the questionnaires were not revealed before opening. The order of the two parts (1–2) was changed midway during the period the survey was open (March 12 to April 29, 2007). Responders were judged to have some (more than average) experience in evidence-based medicine if they reported a frequency of use of external evidence (Medline, Cochrane Library) within the upper half of responses.

The results of the survey were presented using descriptive analysis (mean, median, and standard deviation for perceived clinical relevance and perceived need for evidence). In addition, the proportion of responders considering the intervention very important or extremely important is shown.

### Literature-based data extraction

Reviews dealing with interventions to treat diabetes or diabetic complications, or dealing with the treatment of other disorders but specifically aimed at diabetic patients were identified in the Cochrane Library, issue 1/2008, using the search term diabet* (title, abstract, keywords). Reviews relating to prediabetes, gestational diabetes, or type 1 diabetes were excluded, as were reviews for which the full text was withdrawn and thus not available online anymore. Reviews were evaluated using a standardized form. Items assessed included details of the research question (interventions, comparisons, populations, and outcomes), as well as details on the inclusion criteria (methods of included studies) and the results (such as number and quality of included trials, conclusiveness of the results, adverse events). We also examined time to completion, measured as time from publication of a protocol to time of first publication of a review.

Patient subgroups included, for example, children with diabetes, or patients with obesity, insulin-dependent or insulin-refractory course, co-morbidities (e.g., dyslipidemia, hypertension, cardiovascular problems), acute complications (hypoglycemia, hyperglycemia) and long-term complications (e.g., diabetic foot, polyneuropathy, nephropathy, retinopathy), and patients in other/special situations (e.g., pregnancy, surgery, drug addiction).

Outcomes of reviews were categorized as major (such as mortality, stroke, myocardial infarction, amputation, loss of vision, renal failure/dialysis), intermediate (pain, infection, angina, ulcers, walking distance, episodes of hypoglycemia), performance related (quality of life, days off work, days in hospital, costs) and surrogate (glycemic control (glycosylated hemoglobin A, glucose) or any other measurements in body liquids (hormones, lipids, creatinine etc), weight/body mass index or blood pressure).

The conclusiveness of the results of a review was categorized as conclusive (recommendation for or against, equivalence of treatments), somewhat conclusive (potential recommendation, recommendation for subgroups only) and inconclusive (“uncertain”, “more evidence needed”).

### Ethics

The survey was classified by the local ethics committee as an evaluation among professionals and as such exempt from ethical consent. Participation was anonymous and on a voluntary basis. The evaluation of reviews was based on published materials not containing individual patient data.

## Results

### Survey participation

There were 459 responders, rather equally distributed between the two parts of the survey (229 for perceived clinical relevance/230 for perceived need for evidence, Table 1). Physicians specializing in the care of diabetes, as board-certified by the German Diabetes Society (www.ddg.de), constituted about 50% of all respondents, followed by specialists in internal medicine/endocrinology and specialized nurses and support staff. There were few general practitioners or other doctors without specific diabetes training in the sample.

**Table 1:** Survey participants.

	Part of questionnaire	
Professional group	perceived clinical relevance	perceived need for evidence
Physicians		
Diabetes specialist	107 (51%)	99 (47%)
Endocrinologist, Internist	22 (10%)	46 (22%)
Other specialist	3 (1%)	0
G.P. without diabetes focus	3 (1%)	10 (5%)
not specialized/in training	10 (5%)	9 (4%)
Other		
Diabetes nurse/counselor	46 (22%)	32 (15%)
Other professional	19 (9%)	14 (7%)
Patient/relative, consumer	0	2 (1%)
Total number of respondents	229	230

Item response varied and was generally lower for rare interventions (e.g., midodrin in autonomic hypotension, item response: 137/229; 60%) or new interventions (e.g., exenatid, year of EMEA approval 2006, item response 150/230; 66%). For most items, there were between 170 and 179 respondents. There were no differences in item response rates by professional group, experience in evidence-based medicine or part of questionnaire (clinical relevance or need for evidence), but item response dropped towards the end of the questionnaire.

### Results of Cochrane Review assessment

Of 105 reviews identified using the search term diabet* in the Cochrane Library, 56 completed systematic reviews were found to be eligible. The remaining reviews did not specifically deal with diabetes, diabetic patients, or diabetic complications (n = 41), or full texts were unavailable (withdrawn reviews, n = 8). In addition, there were 48 protocols for reviews which would be eligible once completed. The reviews included, on average, 17 studies (range: 0 to 205, IQR 23 studies). The majority of reviews exclusively dealt with randomized controlled trials only (43 reviews, 77%). Quasi-randomized trials were included in 9 reviews (16%), non randomized trials in 4 reviews (7%), and observational trials in 9 reviews (16%) (categories not mutually exclusive) (Figure S1). Generally, the inclusion of non randomized and observational studies, either exclusively or along with randomized trials, was a feature of reviews on complex interventions and lifestyle modification; it was not found to occur in reviews on drug interventions.

Mostly, the quality of included studies was judged by the review authors to be variable (in 19 reviews) or poor (in 20 reviews), in only 1 review (2%) the quality was reported to be good. In 17 reviews, the authors did not proceed to carry out meta-analysis (i.e., there was no statistical pooling of the study results), either due to lack of studies or due to insufficient quality or due to a high degree of heterogeneity (author decision). The median time to completion was 18 months (maximum 120 months). The results of the meta-analyses were conclusive in 22 reviews (39%), somewhat conclusive in 25 reviews (44%), and inconclusive in 9 reviews (16%). All conclusions incorporated a statement that more trials were needed (100%).

### Survey results and corresponding systematic reviews

Lifestyle modifications (10 items) were generally considered clinically very relevant in the management of diabetes (Table 2). In particular, physical activity received a mean score of 4.5 and was considered very important. In contrast, interventions to reduce salt content, the use of food additives, drug interventions, and surgical procedures to achieve weight loss were not considered important. The perceived need for evidence was generally similar to the perceived clinical relevance in this group. However, for drug interventions or surgical procedures to achieve loss of weight, although considered not important (drug: 2.1 (SD 0.77); surgical 1.9 (SD 0.67)), a relatively high need of evidence was perceived (drug 3.37 (SD 1.10); surgical 3.0 (SD 1.26)).

**Table 2:** Life style interventions and weight reduction, mean values and percentage attaching high values (%).

Intervention	Perceived relevance				Perceived need for evidence				R	P
	N	mean	SD	n* (%)	N	mean	SD	n* (%)		
Physical Activity	179	4.47	0.61	168 (94%)	171	4.10	1.00	138 (81%)	3	1
Weight reduction in general	178	4.17	0.88	146 (82%)	175	3.93	0.98	129 (74%)	4	1
Calory reduction	177	3.89	0.88	117 (66%)	172	3.66	1.05	104 (60%)	0	0
Fat reduction	178	3.70	0.94	107 (60%)	170	3.50	1.01	97 (57%)	1	1
Alcohol/nicotine abstinence	177	3.59	0.93	93 (53%)	169	3.30	1.17	72 (42%)	0	0
Carbohydrate intake modification	177	3.24	0.92	69 (39%)	174	3.67	0.93	102 (59%)	1	2
Salt reduction	177	2.67	0.91	29 (16%)	173	2.96	1.03	52 (30%)	0	0
Food additives	175	1.65	0.78	7 (4%)	171	2.69	1.20	46 (27%)	1	1
Drug interventions for weight reduction	177	2.12	0.77	10 (6%)	171	3.37	1.10	81 (47%)	2	0
Bariatric surgery	178	1.90	0.67	3 (2%)	171	2.98	1.26	60 (35%)	3	0

* number and proportion of respondents considering the interventions as (highly) relevant or/and in high need of evidence.

N: number of overall respondents; n(%): number of respondents (proportion) considering the relevance/need as very high or extremely high.

R: number of reviews (completed); P: number of protocols (planned reviews) in the Cochrane Library.

The group of interventions of lifestyle modification and weight loss were represented by seven Cochrane reviews, dealing with weight loss in general, fat content modification, carbohydrate content modification, physical activity, dietary supplements, and medical and surgical weight loss interventions. Most reviews used surrogate parameters such as lipids or weight loss as outcomes. Major endpoints (mortality, major morbidity) were examined in only two reviews.

Complementary and alternative interventions (CAM, 5 items) were considered to be rather unimportant: mean scores ranged from 1.5 (9/167; acupressure) to 1.9 (3/169; herbal medicines) (Table 3). The need for evidence was perceived as slightly higher as compared to the perceived relevance, but generally also quite low. In the Cochrane Library there is currently one review available on CAM in a diabetes-related problem, examining the effect of a Traditional Chinese Medicine intervention on glucose metabolism.

**Table 3:** Complementary and alternative methods interventions.

Intervention	Perceived relevance				Perceived need for evidence				R	P
	N	mean	SD	n* (%)	N	mean	SD	n* (%)		
Naturopathy, herbal preparations	167	1.87	0.90	9 (5%)	173	2.50	1.15	31 (18%)	0	0
Traditional Chinese Medicine	168	1.69	0.86	6 (4%)	171	2.28	1.19	31 (18%)	1	4
Acupuncture	170	1.64	0.77	5 (3%)	173	2.18	1.14	28 (16%)	0	1
Homeopathy	168	1.63	0.79	4 (2%)	172	2.22	1.15	25 (15%)	0	0
Acupressure	169	1.54	0.68	3 (2%)	173	1.95	1.05	19 (11%)	0	1

* number and proportion of respondents considering the interventions as (highly) relevant or/and in high need of evidence.

N: number of overall respondents; n(%): number of respondents (proportion) considering the relevance/need as very high or extremely high.

R: number of reviews (completed); P: number of protocols (planned reviews) in the Cochrane Library.

Of the oral antidiabetic drugs, metformin was among the highest rated interventions for relevance as well as for need for evidence (4.4; 4.0) (Table 4). Characteristic for this group of interventions, is the frequent use as comparative treatment (standard therapy) in the evaluation of other therapies. For example, while there is no review to examine the efficacy of sulfonyl urea as a primary study question, this treatment is used as the comparison treatment in 5 reviews. All reviews on oral diabetics included major outcomes.

**Table 4:** Oral antidiabetics and new medications.

Intervention	Perceived relevance				Perceived need for evidence				R	P
	N	mean	SD	n* (%)	N	mean	SD	n* (%)		
Metformin	163	4.35	0.66	148 (91%)	167	3.99	1.04	130 (78%)	5	5
Thiazolidinediones	160	3.36	0.96	76 (48%)	168	4.22	0.84	140 (83%)	5	4
Sulfonylureas	159	3.26	1.07	68 (43%)	167	3.92	1.00	124 (74%)	5	4
Meglitinides	159	3.08	0.99	55 (35%)	169	4.02	0.88	128 (76%)	5	4
Alpha-glucosidase inhibitors	161	2.22	0.99	17 (11%)	170	3.62	1.14	104 (61%)	4	3
GLP analogues	135	3.19	0.94	52 (39%)	158	4.37	0.71	141 (65%)	0	2
DPP 4 inhibitors	130	3.09	0.92	42 (32%)	155	4.33	0.73	135 (87%)	0	0
Amylin analogues	126	2.79	0.98	26 (21%)	154	4.18	0.88	124 (81%)	0	1

* number and proportion of respondents considering the interventions as (highly) relevant or/and in high need of evidence.

N: number of overall respondents; n(%): number of respondents (proportion) considering the relevance/need as very high or extremely high.

R: number of reviews (completed); P: number of protocols (planned reviews) in the Cochrane Library.

More recently introduced preparations are glucagon-like-peptide analogues (GLP; exenatid, EMEA approval 2006), dipeptidylpeptidase-4 inhibitors (DPP-4-inhibitors, sitagliptin, EMEA approval 2007) and amylin analogues (pramlintide, EMEA approval 2005). All were considered of limited to moderate clinical relevance, as yet (3.2; 3.1; 2.8) but in high need of evidence (4.4; 4.3; 4.2) (Table 4). In the Cochrane Library there were, at the time of the survey, no completed reviews on these new interventions. However, 3 protocols were published (GLP-analogues: 2, DPP-4 inhibitors: 1).

Of the different insulins (12 interventions), humane preparations (4.2) were considered clinically somewhat more important than the analogues (short acting, 3.9, long acting 3.7) (not shown). In contrast, the perceived need for evidence was highest for both short and long acting analogues (4.3; 4.0; humane preparations 3.8). A discrepancy between perceived relevance and perceived need for evidence was most pronounced for inhaled insulins (1.7; 3.3). In the Cochrane Library, there were 5 completed reviews on insulins. All used only surrogate and intermediate endpoints (glycemic control, hypoglycemic episodes). Respondents also felt that injection techniques and injection to meal interval were very important, both clinically and in need of evidence. There are, however, no reviews or protocols on these issues.

Of the interventions used to correct dyslipidemia in diabetics (6 interventions), only statins were considered both clinically highly important (4.1) as well as in need of evidence (3.9). For other preparations, the need for evidence clearly superseded clinical relevance. Similarly, for the treatment of hypertension (6 interventions), one class was considered clinically particularly important, as well as in need of evidence (ACE inhibitors/AT1 antagonists: 4.4; 4.1). Beta-blockers, diuretics, calcium antagonist,s and “other antihypertensives” all ranged between 3.1 to 3.9 for both relevance and need for evidence. Salt restriction was not considered important (2.7, perceived need for evidence: 3.2; see lifestyle interventions). In the Cochrane Library, there was, at the time of the survey, no completed review on dyslipidemia (1 protocol), there were 2 reviews on the treatment of hypertension in diabetics and none on other cardiovascular problems (14 interventions, 1 protocol).

Of the complications, the diabetic foot (12 interventions) was considered most important, in particular with respect to the treatment of infections (4.7), regular specialist foot care (4.6) and curative surgical interventions such as debridement and revascularization (4.0). Walking exercises, skin replacement and physical therapy were ranked between 3.0 and 3.9 for both clinical relevance as well as need for evidence. In contrast, drug treatment such as rheologics (2.9), prostaglandins (2.4) and growth factors (2.1) were not considered important in the care of patients. These interventions all scored in the intermediate range for need for evidence. The importance of the diabetic foot is reflected by 7 completed reviews in the Cochrane Library. Generally, intermediate outcomes were used as outcome criteria for this group of interventions, e.g., reduction of pain or adverse events.

The treatment and prevention of neuropathic complications encompasses a large range of different interventions (26 interventions identified). Treatments for autonomic neuropathy (e.g., treatment of erectile dysfunction, gastro paresis or orthostatic dysregulation) were mostly rated in the lower ranges of clinical relevance. E.g., 5-phosphodiesterase inhibitors for erectile dysfunction received a mean value of 2.7, alpha liponic acid 2.4 or anticholinergics for orthostatic dysregulation 2.2. There were only a few exceptions: antiepileptics for peripheral neuropathy (3.3), medical treatment of glaucoma (3.6) and physical maneuvers to prevent neuropathic ulcers (taping/bandages 3.6, positioning of the food 3.3). The need for evidence, in contrast, was generally high in this group, in particular with respect to the different drugs used. Evidence for the treatment of neuropathic complication is limited – there are 7 Cochrane reviews covering topics such as bandaging, antiepileptics, tricyclics, and 5-phosphodiesterase inhibitors in diabetes.

Interventions to treat patients with diabetic nephropathy were generally considered of high clinical relevance, in particular the treatment of hypertension in patients with nephropathy (4.9), and, more specifically, use of ACE-inhibitors in diabetic nephropathy (4.5). The perceived need for evidence correlated well with the perceived relevance. This was true for drug-treatments (ACE inhibitors, relevance 4.9, need for evidence 4.3), as well as dietary restrictions (protein-restriction, 3.4; 4.5), transplantation (3.6; 3.8) and hemodialysis (4.0, 3.7). The high perceived relevance of this complication (7 interventions) was not reflected in the Cochrane Library – there are only 2 completed reviews available (ACE inhibitors, protein-restriction) (4 protocols). Both included major outcomes as well as surrogates.

Similar to interventions in nephropathy, interventions in retinopathy were also considered both important and in need of evidence. In the Cochrane Library, there are currently 2 completed reviews on retinopathy (drug treatments of retinopathy). Of psychiatric complications and co-morbidity, treatment of associated dementia and associated depression were considered clinically relevant as well as in need of evidence. The former is covered by a Cochrane review.

The results for acute diabetic complications are presented in Table 5. In hypoglycemia, rapid substitution of glucose was of eminent clinical importance (4.8), need for evidence was perceived as less prominent (3.2). In contrast, bicarbonate infusion and phosphate are considered least important and their need for evidence intermediate. There are neither completed Cochrane reviews, nor protocols on acute care interventions in diabetes.

**Table 5:** Interventions in acute diabetic complications.

Intervention	Perceived relevance				Perceived need for evidence				R	P
	N	mean	SD	n* (%)	N	mean	SD	n* (%)		
**Hyperosmolar coma:**										
i.v. fluids	136	4.77	0.47	133 (98%)	116	3.62	1.23	67 (58%)	0	0
electrolytes (monitoring, substitution)	135	4.61	0.60	127 (94%)	116	3.59	1.22	67 (58%)	0	0
i.v. insuline	136	4.57	0.65	128 (94%)	117	3.46	1.28	66 (56%)	0	0
treatment of infection	135	3.89	0.90	94 (70%)	117	3.34	1.12	55 (47%)	0	0
bicarbonate	134	3.04	1.13	46 (34%)	117	3.35	1.22	54 (46%)	0	0
phosphate	133	2.61	1.04	27 (20%)	116	3.03	1.11	38 (33%)	0	0
**Hypoglycaemia:**										
carbohydrates, glucose	134	4.81	0.48	134 (100%)	119	3.24	1.40	61 (51%)	0	0
glucagone	136	3.76	1.01	83 (61%)	118	3.25	1.29	57 (48%)	0	0

* number and proportion of respondents considering the interventions as (highly) relevant or/and in high need of evidence.

N: number of overall respondents; n(%): number of respondents (proportion) considering the relevance/need as very high or extremely high.

R: number of reviews (completed); P: number of protocols (planned reviews) in the Cochrane Library.

Educating patients in diabetes, diabetes management in general practice, and general organization of diabetes management were considered highly important clinically, as well as in need of evaluation. Generally, training and management issues, both specific (such as perioperative care, vaccinations, screening) and at the level of integrated care are somewhat more difficult to define, so these may have been underrepresented in the survey. There are currently 16 reviews dealing with these types of intervention and 10 protocols.

## Discussion

In this study we explored how caregivers in type 2 diabetes perceive the relevance and need for evidence for therapeutic interventions, as assembled from clinical practice guidelines and expert opinion, and contrasted this to the topics already covered by systematic reviews. For most interventions, the content of the Cochrane Database of Systematic Reviews was well in accordance with the perceived need. Generally, we found that perceived relevance and perceived need for evidence were similar.

Most reviews examined patient-relevant endpoints. There are a few notable exceptions, identifying a need for improvement. In particular, this relates to the immediacy of the reviews, the consideration of specific subgroups and situations, and the inclusion of major endpoints in some interventions, notably insulin analogues.

Deficient immediacy is a common problem in Cochrane reviews [19]–[20]. We found a median time from protocol completion to the publication of the review of 18 months. Overall, the preparation of a systematic review may well take two years or longer. This is certainly a point the Cochrane Collaboration needs to work on in order to better meet the needs of clinicians, in particular with respect to new drugs. A survey undertaken among survey Cochrane authors to identify problems in the conduct of systematic reviews may be of help to target issues leading to delays [21].

The quality and usefulness of Cochrane reviews depends on the endpoints (and quality in general) of the clinical trials available for inclusion. Most obvious in this survey was a failure to examine major endpoints in studies on insulin analogues. Also, the very low proportion of studies considered of good quality was striking. However, other than often perceived, most reviews arrived at a conclusive statement. This is in accordance with a previous report from Cochrane reviews in neonatology [22].

The findings of this review are corroborated by a number of strengths. We were able to collect opinions from a large group of persons involved in the daily care of persons with diabetes, covering a variety of professional groups. The similarity of opinions across the different groups was striking, evidencing a high generalizability of the findings on a national level even though estimates as to the representativeness of the sample are difficult to provide. As there is no standard way to assess what care givers would like to see reflected in systematic reviews, we had chosen two complementary approaches in the survey: asking for relevance, and asking for perceived need for evidence. The difference between these two approaches was particularly evident in the extreme situations of acute emergencies and new drugs: Giving glucose in hypoglycemic shock was considered clinically extremely important but less in need of evidence – this is akin to the notorious parachute example [23].

There are several limitations. First, this was a national survey, at best representative for the perceptions of care givers in Germany. Perceptions may differ in other countries. We did not seek the opinions of consumers (patients with diabetes, family members) or clinician-patient cooperation in priority setting, as is the focus in several recent projects [14], [24], [25]. Similarly, the views of members of health care management organizations and administrational bodies were not sought. Our survey was explicitly restricted to professional care givers in the medical community. Also, there are some limitations based on the design of the study. Perceived clinical relevance and perceived need for evidence were rated by different persons, randomly self-assigned. The concepts were not specifically defined or explained to the respondents in order to elicit reactions as spontaneous as possible. There may have been different concepts of “need for evidence” in different respondents.

Lastly, diabetes is but one topic in the Cochrane Collaboration, and Cochrane reviews comprise only about 20% of systematic reviews available in the literature. Most of the reviews identified were edited by the Cochrane Metabolic and Endocrine Disorders Group (http://www.endoc.cochrane.org/de) and follow highly standardized forms prepared by this group. Specific organ complications fall into the responsibilities of various other Cochrane editorial groups, such as diabetic nephropathy in the Kidney Group, and diabetic retinopathy in the Eyes and Vision Group. Therefore, our finding that patients suffering from late diabetic complications seem to be underrepresented in Cochrane Reviews, as compared to the ascribed clinical relevance, may be a consequence of the specific infrastructure within the Cochrane Collaboration. This refers to issues of overlap in the responsibilities of various Cochrane editorial groups. It may not apply to other disease entities.

In summary, in general, we found diabetic interventions well covered in the Cochrane Library with some areas for improvement highlighted. We would like to encourage researchers to acknowledge the priorities set by clinicians and other medical care givers.

## Supporting Information

Figure S1 **Characteristics of included Trials.** y-Axis: Percentage of all included Trials (not mutually exclusive).
(TIF)

Table S1 **Full list of interventions.**
(DOC)
