# Supplementary figures and images for: Prioritisation of Clinical Research by the Example of Type 2 Diabetes: A Caregiver-Survey on Perceived Relevance and Need for Evidence
Source: PLoS One. 2012 Mar 20;7(3):e32414. doi: 10.1371/journal.pone.0032414 (PMC3308957; doi:10.1371/journal.pone.0032414)

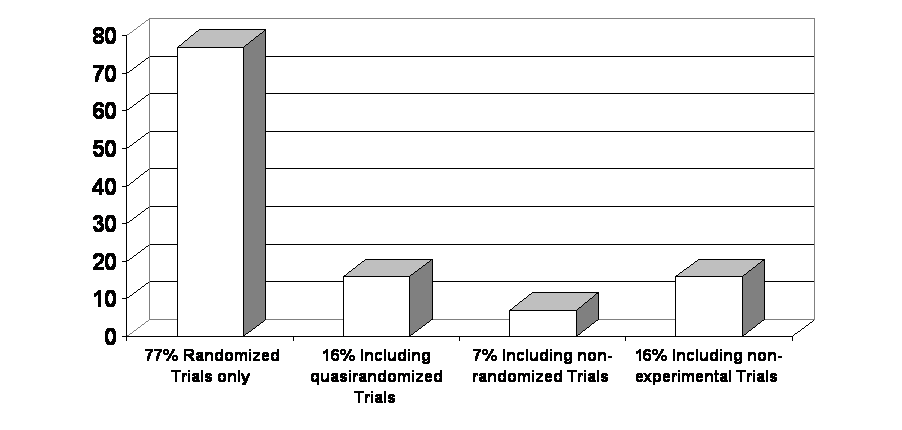

Supplement: Figure S1 — Characteristics of included Trials. y-Axis: Percentage of all included Trials (not mutually exclusive). (TIF) [file pone.0032414.s001.tif]
