# Supplementary material for: Prioritisation of Clinical Research by the Example of Type 2 Diabetes: A Caregiver-Survey on Perceived Relevance and Need for Evidence
Source: PLoS One. 2012 Mar 20;7(3):e32414. doi: 10.1371/journal.pone.0032414 (PMC3308957; doi:10.1371/journal.pone.0032414)
Supplement: Table S1 — Full list of interventions. (DOC) [file pone.0032414.s002.doc]

Full list of interventions

| **Life style interventions and weight** **reduction**:  Physical Activity  Weight reduction in general  Calory reduction  Fat reduction  Alcohol / nicotine abstinence  Carbohydrate intake modification  Salt reduction  Food additives  Drug interventions for weight reduction  Bariatric surgery  **Complementary and alternative methods interventions**:  Naturopathy, herbal preparations  Traditional Chinese Medicine  Acupuncture  Homeopathy  Acupressure  **Oral antidiabetics and new medications**:  Metformin  Thiazolidinediones  Sulfonylureas  Meglitinides  Alpha-glucosidase inhibitors  GLP analogues  DPP 4 inhibitors  Amylin analogues |
| --- |

| **Insulintherapy** :  Animal Insulin  Humaninsulin  quick-acting Insulin  long-acting and intermediate Insulin  long-acting Insulin and oral Antidiabetics  mixed Insulin  conventional Therapy  intensive Therapie  Insulinpumptherapy  inhaled Insulin  Timing of Insulinapplication  Injectiontechnique  GLP-Analogues  Amylin-Analogues  DPP4-Inhibitors  **Medical Treatment of Dyslipidaemia**:  Statine s  Fibrate s  nicotinic Acid  Colestyramine  Ezetimibe  omega-3-fatty Acids  **Treatent of cardiovascular Disease:**  Vitamines and trace Elements  Betablockers  ACE-Inhibitors / AT1 Antagonists  Calcium Antagonist  Diuretics  other Antihypertensives  Sodiumrestriction  postmenopausale Hormontherapy  Thrombolysis  PCI  Aspirin / Clopidogrel / Ticlopidin  low-molecular Heparin  Heparin  Vitamin K Antagonists |
| --- |
| **Treatment of the diabetic** **foot**:  podological Care  physical Therapy  Physiotherapy  rheological Interventions  PGE1-Application  local Application of Growthfactors  Revascularisation  Skin-Replacement  Woundcare  **Surgical Treatment of the diabetic** **foot**  Amputation  Treatment of diabetic Neuropathy  Casts  Alpha-Lipoic Acid  NSAR  Opioids  Antiepileptics  Mexiletin  tricyclic Antidepressivs  Capsaicin  Fludrocortison  Bloodpressure-affecting Drugs  Procinetics  parenteral Nutrition  gastral Elektrostimulation  Antidiarrhoics  Laxantives  Biofeedback  Autokatheterisation  Parasympathicomimetics  phosphodiesterase Inhibitors  Aprostadil  rectile-Support Devices  Anticholinergics  Clonidin  topic Therapy  Treatment of Glaucoma |
| **Treatment of diabetic** **Nephropathy**:  Bloodpressure-Management  Sodium- / Proteinrestriction  ACE-Inhibitors  Treatment of urinary Tract Infection  Electrolytemanagement  pH-Level-Management  Vitamin D Substitution  Therapy of renal Anaemia  peritoneal Dialysis / Haemodialysis / Haemofiltration  Nephrotransplantation    **Treatment of diabetic** **Retinopathy**:  Angiogenesisinhibitors  Laserphotocoagulation  Vitrectomie  Catarakt-Extraction  Treatment of Glaucoma    **Treatment of neuropsychiatric** **Disorders**  Treatment of Dementia  Treatment of Depression  **perioperative Diabetes**-**Management** :  i.v. – Glukose / Insulin / Potassium - Substitution  **complex Interventions**:  Outpatient-Care  Fluvaccination / Pneumococcal vaccination  **Treatment by medical Specialist**:  Retinopathyscreening  dental Care  Screening for vascular Disease  Patienttraining  Diabetesmanagement  psychological Interventions |

| **Hyperosmolar coma**:  i.v. fluids  Electrolytes (monitoring, substitution)  i.v. insuline  Treatment of Infection  Bicarbonate  Phosphate  **Hypoglycaemia**:  Carbohydrates, Glucose  Glucagone |
| --- |
